# Drought Analysis of the Haihe River Basin Based on GRACE Terrestrial Water Storage

**DOI:** 10.1155/2014/578372

**Published:** 2014-08-18

**Authors:** Jianhua Wang, Dong Jiang, Yaohuan Huang, Hao Wang

**Affiliations:** ^1^State Key Laboratory of Simulation and Regulation of Water Cycle in River Basin, Department of Water Resources, China Institute of Hydropower & Water Resources Research, Beijing 100038, China; ^2^Institute of Geographical Sciences and Natural Resources Research, Chinese Academy of Sciences, Beijing 100101, China

## Abstract

The Haihe river basin (HRB) in the North China has been experiencing prolonged, severe droughts in recent years that are accompanied by precipitation deficits and vegetation wilting. This paper analyzed the water deficits related to spatiotemporal variability of three variables of the gravity recovery and climate experiment (GRACE) derived terrestrial water storage (TWS) data, precipitation, and EVI in the HRB from January 2003 to January 2013. The corresponding drought indices of TWS anomaly index (TWSI), precipitation anomaly index (PAI), and vegetation anomaly index (AVI) were also compared for drought analysis. Our observations showed that the GRACE-TWS was more suitable for detecting prolonged and severe droughts in the HRB because it can represent loss of deep soil water and ground water. The multiyear droughts, of which the HRB has sustained for more than 5 years, began in mid-2007. Extreme drought events were detected in four periods at the end of 2007, the end of 2009, the end of 2010, and in the middle of 2012. Spatial analysis of drought risk from the end of 2011 to the beginning of 2012 showed that human activities played an important role in the extent of drought hazards in the HRB.

## 1. Introduction

Driven by global change and population pressure, droughts are one of the most serious natural hazards [1] that can lead to crop losses and economic havoc in many areas. For example, in China, the direct economic loss associated with droughts in 2011 was up to 102.8 billion Yuan [2]. Various global research projects, including ISCCP, IHDP-IRG, and CLIVAR, have included drought research as an important part of their research plans. For basin-scale drought monitoring and evaluation, an understanding of the spatiotemporal variation pattern of water deficit is needed.

A drought is regional by nature and it is characterized by its total water deficit (including surface water, biological water, soil water, and ground water or snow/ice). However, quantifying total water deficit over large areas is a major challenge in drought studies unfortunately; conventional data resources are not sufficient in TWS evaluations for monitoring drought occurrence, extent, and intensity on a regional scale. First, in situ meteorological and hydrological measurements are limited in both space and time, because they are point measurements that cover a small region around the gauging station. Globally, or even at a basin scale, in situ monitoring networks that include all TWS parameters are incomplete [3], which leads to a shortage of sufficient and precise data in vast regions. Furthermore, other parameters (e.g., evapotranspiration) are measured indirectly, which may reduce the accuracy of TWS estimation in drought estimation. Second, hydrological and climate models based on in situ measurements are valuable for estimating the distribution of changes in TWS, while the need for various inputs of land surface parameters (e.g., land cover), which are always difficult to observe and hasve increased the uncertainty of the TWS simulation in drought estimation [4, 5]. In addition, the modeling results for of the TWS were insufficient for evaluating severe extreme droughts and climate events such as droughts [6]. Lastly, conventional optical remote sensing and altimetry based measurements, which have been proven to be helpful for water balance studies at basin scales [7]. However, they are also problematic in drought evaluation. They are limited in detecting land-water changes several centimeters below the surface of the earth, which are just some of the components needed for TWS estimation. For example, it is not valid for evaluating groundwater, which is essential for drought monitoring [8, 9].

Due to the difficultly of measuring the integrated bulk variables of TWS at basin scales, recent drought evaluations have mainly relied upon subcomponents (e.g., precipitation) or proxies (e.g., NDVI, CWSI) [10, 11], such as the four drought categories of “meteorological,” “hydrological,” “agricultural,” and “socioeconomic” [12]. However, drought indicators rely upon these proxies and approximations are not representative to quantify the integrated temporal variations of water resources at basin scales. Considering that the horizontal water cycle at the basin scale, surface flow, interflow, and ground water flow from upstream basins may alleviate droughts in downstream basins, drought severity will be overestimated when based only on proxies for precipitation. In the vertical view, the water cycle processes of infiltration and evapotranspiration for surface water, soil water, and groundwater also affect the proxies used for drought evaluation. For example, the soil moisture or vegetation index proxies will ignore the initial drought due to the surface water supply of deeper water resources. In addition, using indices based on surface water (rain fall, flow rates) may underestimate the severity of effects in the later stage of droughts for the deep water recharge from surface water. For regions with prolonged drought conditions, deep soil water and ground water may be more suitable for evaluating droughts [13]. Furthermore, droughts are becoming more complicated due to anthropogenic impacts. For example, irrigation for agriculture will increase precipitation through evaporation and transpiration [14], which is in contrast to the result of irrigation leading to reductions in regional water resources [15]. It has also been shown that using proxies for evaluating long time-series droughts is problematic. Herewith, the only way to evaluate droughts at the basin scale is through an integrated measure of TWS [3].

The gravity recovery and climate experiment (GRACE) is the first dedicated satellite gravity mission that was jointly launched by the National Aeronautics and Space Administration (NASA) and the German Aerospace Center (DLR) in March 2002 [16]. At the basin scale, GRACE provides a new data source for measuring integrated water storage change on time scales ranging from months to decades [17]. The precision of the GRACE-retrieved TWS has been verified in different basins around the world [18–21]. Several studies have used GRACE satellite data to monitor TWS depletion at large river basin scales during droughts [3, 6, 8, 9, 22–24]. Some of them have been used in actual drought monitoring [8] (http://drought.unl.edu/MonitoringTools.aspx). Overall, drought evaluations based on GRACE still need further research. Furthermore, few studies have investigated the distinction between drought monitoring results using GRACE-derived TWS and other proxies in the basins of China.

**Figure 1:**
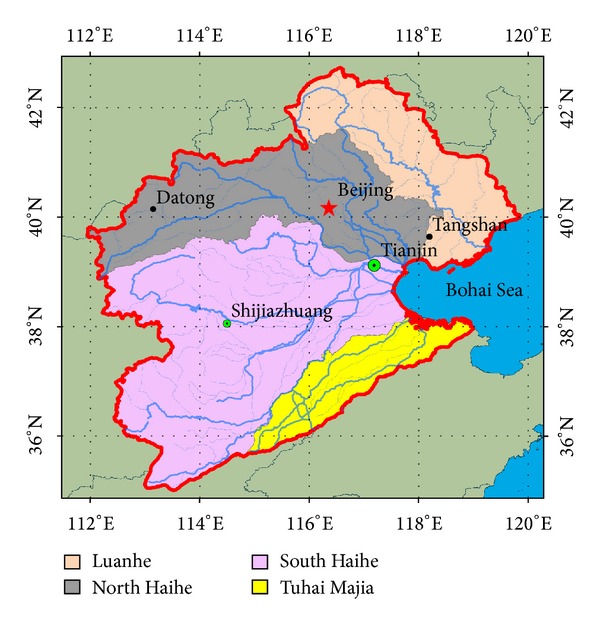
Location of the Haihe River basin with its main subbasins.

In the present study, we use three types of observational data to characterize a multiyear drought in the HRB of China (see Figure 1 for location): the GRACE-derived TWS, precipitation, and the vegetation index. Rather than considering the HRB as a whole, as has been done in most studies, we analyzed the spatiotemporal variability patterns of droughts detected by GRACE-derived TWS and the other two proxies along the main river, from upstream to downstream, as well as along its main tributaries. The results of this paper can be used to investigate the consistency between GRACE-derived TWS changes and droughts of China at the basin scale.

## 2. Study Area and Data

### 2.1. Location and Hydrology of the HRB

The HRB is the fourth basin in China, which lies in North China from 34°09′N to 43°11′N and from 111°21′E to 120°43′E. The total area of the HRB is more than 318,000 km^2^, and approximately 40% of the area is plain, while 60% is mountainous. The elevation of the basin, which ranges from higher than 2900 m to below 3 m, decreases from the Yunzhong and Taiyue mountains to the West to the littorals of the Bohai Seain the East [25]. The HRB is dominated by a semimoist and semiarid continental monsoon climate with cold, dry winters and hot, humid summers. The spatiotemporal distribution of precipitation within the basin is uneven, with a multiyear average of 550 mm/year, which is the lowest along the East coast of China. Most of the precipitation is temporally concentrated in July and August and spatially concentrated along the coast and the windward side of the mountains [26]. On longer time scales, precipitation analysis has detected a decreasing trend from 1961 to 2010 of −1.7%, which is the largest among the ten major river basins in China [27]. Most studies have projected significant reductions in precipitation, evapotranspiration, and runoff together with increased air temperature. As observed in Figure 1, the HRB consists of four parts: the Luan subbasin, the north Haihe subbasin, the south Haihe subbasin, and the Tuhai-Majia subbasin.

The HRB consists of eight provinces, including Beijing, Tianjin, most areas of Hebei, and some parts of Shandong, Henan, Shanxi, Inner Mongolia, and Liaoning. It is the political and economic center of China. The population of the basin accounts for approximately 10% of China and just 3.3% of the geographical area of the nation. According to the census register, the urban population of the basin is more than 36-million, and the rate of urbanization has reached 28.9%. Approximately 15% of the national GDP is concentrated in the basin, which is above the national average. Agriculture is the largest land-use type and accounts for over 90% of the arable lands. The basin is one of the major grain producing areas of China, as it accounts for 10% of the total agricultural output. The basin produces some 30% of the wheat and 20% of the corn in China [28–31]. To meet the demand for water resources, massive amounts of water have to be diverted from the Yangtze and Yellow Rivers to the basin. The combined effects of climate change and human activities have caused the HRB to continually suffer from droughts over the last 30 years [32]. Several severe droughts occurred in HRB in the past century, which significantly restricted its social and economic development.

### 2.2. Data Acquisition

#### 2.2.1. GRACE Data

GRACE gravity satellite program was jointly developed by the National Aeronautics and Space Administration (NASA) of the United States and the German Aerospace Center (DLR) with the objective of providing spatiotemporal variations of the Earth's gravity field. The U.S. Jet Propulsion Laboratory (JPL) is responsible for the project management of the GRACE gravity satellite program. Monthly gravity field solutions are computed at the University of Texas at Austin Center for Space Research (CSR), the German Research Centre for Geosciences Potsdam (GFZ), JPL, Groupe de Recherche de Geodesie Spatiale (GRGS), and the Delft Institute of Earth Observation and Space Systems (DEOS) as well as Delft University of Technology, among others. Originally, the GRACE results were provided in the form of spherical harmonic coefficients of geoid heights at monthly (or submonthly) intervals. At time scales that ranged from months to decades, temporal changes in Earth's gravity field were detected by GRACE and related to the surface mass redistribution of continental water storage [18]. Gridded monthly data from the land, which were expressed as equivalent water height (EWH), were also released. Here we used the most recent release (RL05) of GRACE products prepared by the CSR GRACE science working team (available at http://grace.jpl.nasa.gov/data/gracemonthlymassgridsland/). These are monthly equivalent water height solutions provided as 1° × 1° global grids from January 2003 through January 2013. There are 116 monthly equivalent water height solutions for the land, with four months missing (June 2003, January 2011, June 2011, May 2012). This new data set replaced the degree two order zero and degree one with parameters from previous studies [33, 34]. A spherical harmonic filter cutoff at degree 60 acted as a third filter on the data. The width of the Gaussian filter that was used for product smoothing was 200 m, which was less critical than earlier solutions to improve the destripping procedure [17]. The product of the land also removed a postglacial rebound signal according to the models of Paulson [35]. At our study area location in the HRB, the impact of the postglacial rebound signal was small. To restore much of the energy that removed by destripping approach to the land grids, a grid of multiplicative scaling coefficients was applied to the monthly land GRACE mass grids. The scaling coefficients were derived independently of the GRACE data by applying the same filtering techniques to the modeled TWS data and then computing the signal attenuation at each geographic location [19, 20, 36]. Using the monthly GRACE-TWS data, the TWS anomaly index (TWSI) from January 2003 to January 2013 in the HRB was calculated by removing the means of annual TWS change:

(1) $$\begin{array}{c} \text{TWSI}={\text{TWS}}_{iY}-\bar{{\text{TWS}}_{i}}, \end{array}$$

where TWS_*iY*_ was the TWS change of *i* month of *y* year, $\bar{{\text{TWS}}_{i}}$ was the average TWS change in the *i* month in the normal period, which was calculated from 10 years of data from 2003 to 2013.

#### 2.2.2. Precipitation Data

In this study, a normal index of meteorological droughts of the precipitation anomaly index (PAI) was proposed, which is used to compare with the TWS anomaly index (TWSI) in drought detection. The PAI is calculated from the version 2.0 monthly precipitation data from the China meteorological data sharing service system (CMDSSS) of the China meteorological administration (CMA), which are available at http://cdc.cma.gov.cn/home.do. These data consist of a national gridded time series with a spatial resolution of 0.5° × 0.5° that covers the time span from 1961 to 2013. The data were generated using a “climatological background field interpolation” based on monthly precipitation from 2416 national meteorological stations and resampled DEM. To be consistent with the GRACE estimates, the monthly precipitation data were linearly interpolated to a 1° × 1° grid. The PAI from January 2003 to January 2013 in the HRB was calculated as follows:

(2) $$\begin{array}{c} \text{PAI}=\frac{P_{i}-\bar{P_{i}}}{\bar{P_{i}}}, \end{array}$$

where *P*_*i*_ denotes the precipitation of *i* month, $\bar{P_{i}}$ denotes the average precipitation in the *i* month in the normal period, which is calculated using the 10 years of data from 2003 to 2013.

#### 2.2.3. Vegetation Index Data

The vegetation index data used in this paper were 1 km, 16 days composited MODIS EVI (MOD13A12), which were downloaded from the NASA EOS data Gateway (EDG) (http://modis.gsfc.nasa.gov/index.php). To be consistent with monthly GRACE estimates, the 16 days EVI product was recomposited using the maximum value composite (MVC). The composited monthly EVI data were then linearly interpolated to a 1° × 1° grid. Anomalies in the EVI for the study period were computed based on averages from 2003 to 2013 [37].

(3) $$\begin{array}{c} \text{AVI}={\text{EVI}}_{i}-\bar{{\text{EVI}}_{i}}, \end{array}$$

where EVI_*i*_ was the EVI value of *i* month and $\bar{{\text{EVI}}_{i}}$ was the long term average the EVI of *i* month.

## 3. Results

### 3.1. Spatiotemporal Variability in TWS, Precipitation, and EVI

**Figure 2:**
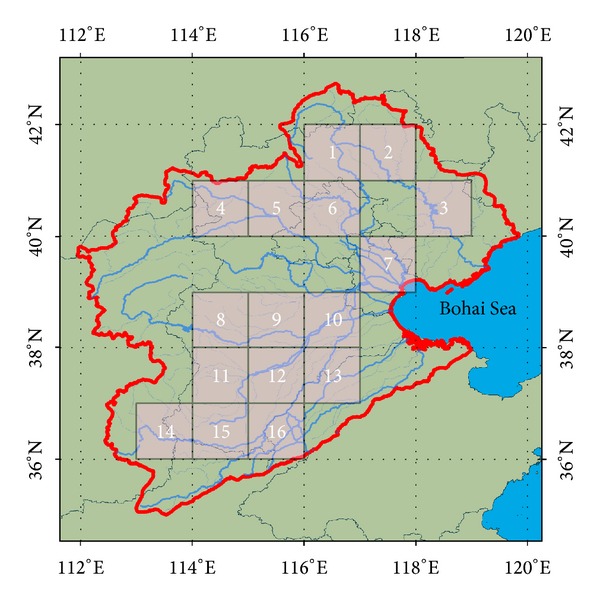
Location of 1 × 1 pixels for drought evaluation using the TWS from GRACE, the precipitation, and the vegetation index.

The time-series total water storage (TWS) change from GRACE, precipitation, and EVI from January 2003 to January 2013 was computed with a 1° × 1° grid resolution along the rivers of the HRB (pixel locations are shown in Figure 2). TWS change was expressed in GRACE equivalent water height. Because the area of Tuhai Majia basin was small compared to the resolution of the 1° × 1° grid, we simply combined it with the South Haihe River basin.

#### 3.1.1. Luanhe River (Pixels 1 to 3 in Figure 3)

**Figure 3:**
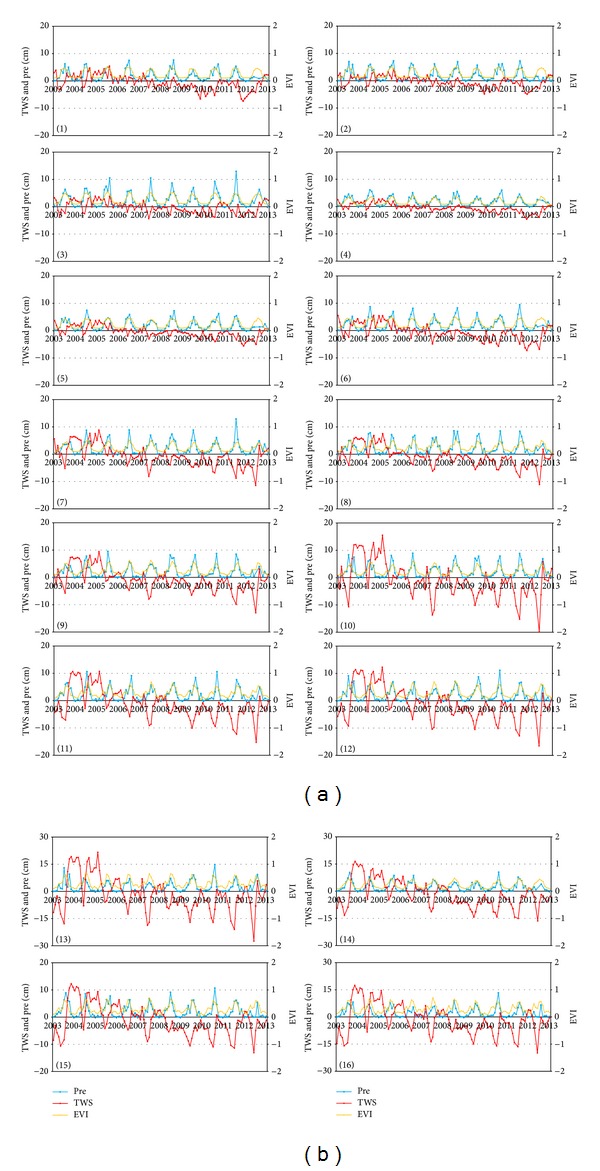
Time-series of TWS, precipitation, and the EVI of selected pixels (the unit of TWS change is cm; the unit of precipitation is 2 cm).

Pixels 1 to 3 in Figure 3 show the temporal evolution of TWS change, precipitation, and EVI in the Luanhe River. In contrast to the annual cycles of precipitation and EVI, the interannual variability in the TWS evolved significantly from 2003 to 2013. Because it is dominated by a temperate monsoon climate, the time-series profile of the EVI shows less interannual variability with a strong seasonal signal peak in summer when the vegetation was flourishing. This indicated that simply using the EVI profile to monitor for droughts is limiting. The time-series profiles for precipitation showed seasonal trends that were similar to those observed for the EVI, with high rainfall during the summer monsoon. For drought monitoring, slight fluctuations in the peak amplitude of the precipitation profile were more evident than with the EVI. However, both profiles lacked significant water deficits associated with droughts. The TWS changes time-series profiles that were collected from GRACE showed the most zigzag water resources diminishment with less of an innerannual cycle, which indicated the extreme hazards associated with droughts. The TWS pixel change from 1 to 3 showed that the water resources in the Luanhe River basin continued to be reduced, with lower than 5 cm of water height increase. Moreover, the area suffered a prolonged period of diminishing water storage from mid-2007 to mid-2012, which may imply that the frequent drought events in recent years may be subject to an extreme, sustained drought for 5 years. The minimum TWS decrease was reached by the end of 2011, which indicated that there may have been a drought event in the Luanhe river basin. A consecutive low value of TWS change and a decrease from the end of 2009 to the middle of 2010 may have also brought about an extreme drought event. Whereas, after a period of decreasing water storage from mid-2007, these regions showed a slight recovery trend, which may indicate the ending of the five-year drought in the Luanhe basin, in pixel 3, which is downstream of the Luanhe River, the amplitude is relatively lower than the two pixels upstream.

#### 3.1.2. North Haihe River (Pixels 4 to 7 in Figure 3)

The figures of pixels 4 to 7 in the North Haihe basin presented similar EVI and precipitation temporal stability patterns to the Luanhe basin. The upstream area of pixels 4 and 5 showed a slight positive TWS anomaly from the beginning of 2003 to mid-2005, where and when droughts were unlikely to occur. Whereas, a period of continuous negative TWS anomaly was observed from the end of 2005 to the beginning of 2013 with a low trough between 2012 and 2013, temporal TWS changes in pixels 6 and 7 fluctuated more significantly, with the largest positive and negative TWS anomalies being more than 5 cm and 15 cm in water height. This indicated that the area downstream had a higher risk for droughts. The figures show that the TWS reductions of pixels 6 and 7 reached a minimum by the end of 2011 and the beginning of 2012 but recovered after mid-2012. This indicated a slight drought event occurred at the beginning of 2012. The time series corresponding to pixel 7 showed large water decrease in the North Haihe river from 2007, which was supposed to be affected by the TWS decrease in the South Haihe river basin and leakage from the ocean.

#### 3.1.3. South Haihe River and Tuhai Majia River (Pixels 8 to 16 in Figure 3)

For narrow shape of the Tuhai Majia Basin by comparing to the resolution of GRACE TWS [2], we combined the South Haihe basin with the Tuhai Majia basin as one region (pixels 8 to 16 in Figure 3). The magnitude of the TWS change of combined sub-basin is larger than the other two sub-basins of Luanhe River and North Haihe River, which indicated a higher potential for floods and droughts in the combined sub-basin. From pixels 8 to 16 in Figure 3, we notice that the temporal variability of TWS evolved significantly. A wet period between 2004 and 2005 was observed with large TWS increases (more than 20 cm EWH in Pixel 13). Whereas, there was no significant increase in precipitation, the TWS increase may be attributed to water transfer from irrigation [38]. After a period of water storage increase, these regions experienced a prolonged decreasing trend from the beginning of 2006 to the beginning of 2012 with nearly negative TWS changes during the whole period. This indicated that these regions may have suffered from an extreme sustained drought for more than 6 years. The time series TWS change profiles clearly indicated several significant TWS deficit peaks, such as mid-2007, mid-2009, mid-2010, mid-2011 and the beginning of 2012. Integrated analysis of time-series TWS change profiles of all 9 pixels implied that extreme droughts may occur at the end of 2007, end of 2009, end of 2010, and in the middle of 2012. The minimum at the beginning of 2012 and the relatively low value of TWS change in all 10 profiles from mid-2011 indicated that there may have been an extreme drought during the 6 years before the middle of 2012. The slight TWS recovery, which was only detected in pixels 9, 10, 13 during the end of 2012, indicated that this 6 years drought will be prolonged in this region. In pixels 10, 13, and 16, which are located downstream, the magnitude of fluctuations were more evident than that in the upstream pixels. Profiles of the three pixels showed that the largest water storage decrease of more than 20 cm EWH occurred at the beginning 2012 and that the maximum amplitude of the other TWS decreasing peaks all exceed 15 cm EWH. Due to the monsoon climate in the study area, there is more precipitation in the regions closer to the sea (for example pixel 3). The abnormal reduction in the TWS downstream, where associated with a small change in precipitation, may have been attributed to human water consumption or agriculture. It indicates that downstream pixels are more likely to suffer from droughts.

### 3.2. Drought Analysis

**Figure 4:**
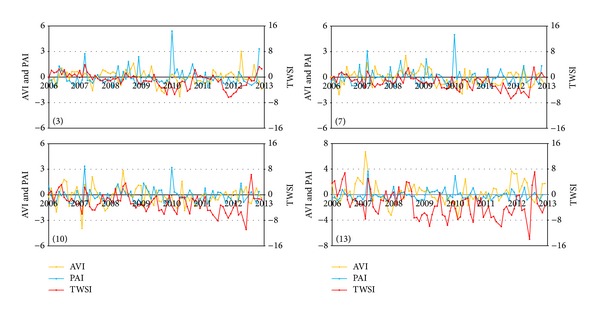
Time-series of the TWSI, the PAI, and the AVI for selected pixels.

Droughts are common in the HRB, and several episodes of potential severe droughts were detected using temporal variability analysis of the GRACE TWS change (Figure 3). To evaluate drought events in the HRB from 2003 to 2013, three indicators were taken into account: the PAI, the AVI, and the annual cycle removed TWS (TWSI). It can be instructive to compare GRACE observations with these common droughts indicators [24, 39]. To compute the TWSI, monthly averaged GRACE TWS change data for ten years were removed at each pixel. Using the spatio-temporal variability analysis described above for the TWS, the precipitation, and the EVI, we chose four representative pixels (pixels 3, 7, 10, 13) from three regions for drought analysis.  Figure 4 shows the time series of the three droughts indicators within the four pixels between 2006 and 2012 when droughts were likely to occur in the HRB.

In contrast to the annual cycle of EVI and precipitation that are shown in Figure 3, temporal variability of the three drought indicators all lack consistency (Figure 4). The correlation coefficients between EVI and precipitation for the four pixels (3, 7, 10, 13) were all more than 0.84 from 2003 to 2013, whereas they were 0.05, 0.07, 0.08, and 0.16 between PAI and AVI, respectively. The low correlation coefficients between PAI and AVI imply that these two drought indicators predict different water resources deficit conditions that accompany droughts. However, correlation analysis showed that both TWS and TWSI present low correlation with EVI, precipitation, and corresponding droughts indicators. The maximum correlation coefficient was 0.34 in pixel 13 between EVI and TWS, which indicated that TWS represented a different drought scene from the two common indicators.  Figure 4 shows that the occurrence and release of drought AVI lagged behind PAI for 1–3 months and droughts of AVI were more severe than PAI. This is because of the delay in recharge of surface and soil water from rainfall and the vegetation growth. Both indicators mainly reflected water depletion in surface water and shallow soil water. For HRB, which included important urban agglomeration areas (Beijing and Tianjin in North Haihe) and agricultural regions (South Haihe and Tuhai Majia), water deficit was the main limitation to maintaining healthy social development [7, 40]. Over the long-term, persistent water shortage in the HRB in recent years has used transferred water and groundwater as the main water supplies. Overexploitation of groundwater caused a depression in the groundwater cone, which has exacerbated the risk for droughts in the HRB [2]. PAI and AVI were both invalid for detecting droughts related to decreases in HRB groundwater where there was a prolonged drought region with strong impacts from human activities. This was most obvious during the period from the end of 2011 to the beginning of 2012 in Figure 4, when the time-series for PAI and AVI had no extreme droughts and a normal and gentle amplitude fluctuation. However, TWSI showed that HRB experienced great water resource decreases during this period, which may have caused an extreme drought in 2006. Considering the annual cycle of precipitation and vegetation growth and their relation to shallow water, the TWS change mainly contributed to the discharge of groundwater to surface water, which implied a drought risk.

**Figure 5:**
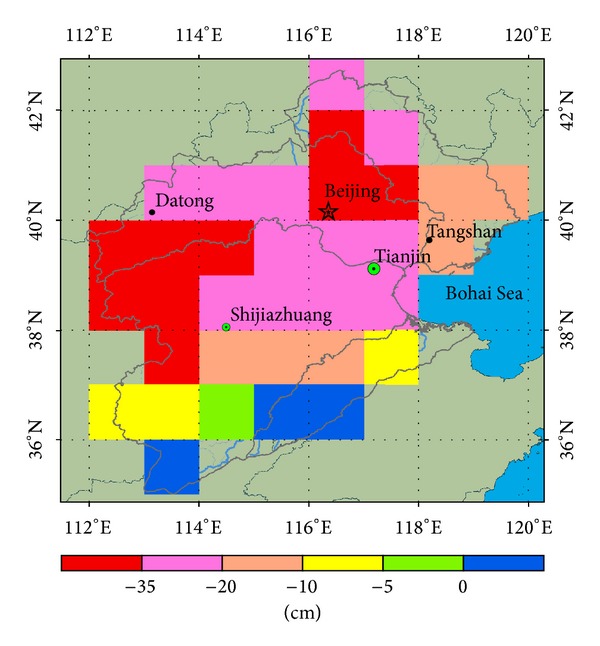
Spatial distribution of accumulated TWS anomalies during the period from October 2011 to March 2012 for drought risk analysis.

To analyze the spatial distribution of droughts in the period between the end of 2011 and the beginning of 2012, we accumulated GRACE TWS anomalies of 6 months from October 2011 to March 2012 in the HRB (Figure 5). As shown in Figure 5, the 6 months of accumulated TWS changes present spatial variability clearly. Most area of the HRB, except a small area of Tuhai-Majia River basin in the southern part of the region, was dominated by drought risk. The droughts in the northern subbasins of the Haihe River basin were more severe with accumulated TWS decreases over an area larger than 20 cm EWH. The drought risk difference in the southern region can be explained by more than 32 × 10^8^ m^3^ of water being transferred annually to the Tuhai-Majia subbasin for irrigation from the Yellow River, which is the second river in China bordering with southern Haihe River basin. Due to the impact of the monsoon climate, there was a trend of higher droughts risk in the western area when compared to the eastern regions near the ocean (Figure 5). The upstream region of the HRB suffered more severe droughts than the downstream region. However, the spatial distribution trend of droughts risk from upstream to downstream was contaminated by scattered urban location pixels. In addition, the accumulated TWS change in pixels around Beijing was lower than those in the upstream region. This azonal distribution was also detected in the areas where the cities of Tianjin, Shijiazhuang, Tangshan, and Datong are located. Therefore, we propose that urban areas with higher domestic and industrial water demand are more susceptible to droughts. The analysis of the spatial distribution of drought risk in the HRB within the selected period indicated that, besides natural climate change, human activity played an important role in drought risk.

## 4. Summary and Discussion

Drought is a recurring issue in many parts of China, including the HRB. In recent years, severe droughts have occurred more frequently over wider regions. GRACE derived vertically integrated water storage (TWS) change, precipitation and the vegetation index have been widely used in quantifying the severity and characterizing droughts at the basin scale in several studies [3, 11, 14]. In this study, we have analyzed droughts by comparing the spatiotemporal evolution of the TWS change, the precipitation, and the vegetation index as well as three corresponding drought indices over the HRB for the time period between January 2003 and January 2013.

The precipitation and EVI time series correlated well with similar seasonal cycles. The slight fluctuations in the peak amplitude of precipitation and EVI profiles limited their direct application in drought monitoring. The TWS change time series showed less of an interannual cycle, which may be more useful to indicate TWS deficits accompanying drought events. While both precipitation and EVI presented the drought conditions of surface water, the TWS had a low correlation to the EVI and precipitation. The GRACE-TWS was more suitable for evaluating prolonged droughts in the HRB region, whose water deficit was mainly dominated by deep soil water and ground water anomalies.

Although the three drought indices of TWSI, PAI, and AVI all detected the drought events in the HRB, the extent and length of the droughts were different. Correlation analysis showed that the correlation coefficients for all three indices were low, which indicated that they represent different water deficit conditions in droughts. Droughts predicted by the TWSI are more prolonged and more severe than those predicted by the other two indices. In addition, the occurrence and release of drought AVI indicated a longer and more severe drought than with PAI. This can also be attributed to the different parts of the integrated water bulks that the three indices represent.

The combined analysis of the spatiotemporal variability of the TWS and the TWSI showed that the overall drought conditions in the HRB began around 2007. The North Haihe River subbasin suffered the longest period of sustained water diminishment for 7 years. An extreme water deficit occurred in the South Haihe River subbasin with the largest EWH decrease during a 6 year period of sustained water reduction. Drought analysis also shows that extreme drought events may have occurred in four periods at the end of 2007, end of 2009, end of 2010, and middle of 2012, which was consistent with drought reports [2]. Furthermore, a slight water recovery at the end of 2012 was detected in the Luanhe and North Haihe subbasin, which may indicate an end to the prolonged droughts.

In the period from the end of 2011 to the beginning of 2012, the drought in the HRB abated with a return to nearly average monthly precipitation and EVI; however, the GRACE TWSI data show that a substantial accumulated bulk water deficit remains in the basin. Spatial analysis of TWSI drought during this period showed that drought risk in the HRB was impacted both by the natural climate and human activities. Due to the dominating temperate monsoon climate, the downstream regions near the ocean are less threatened by droughts. However, droughts in urban areas will be more severe due to their higher domestic and industrial water consumption. The agricultural areas, such as the Tuhai-Majia River basin, will suffer fewer extended droughts due to water transfer for irrigation.
